# Right atrial volume indexed by cardiovascular magnetic resonance as a predictor of mortality in patients with heart failure with reduced ejection fraction

**DOI:** 10.1186/1532-429X-18-S1-O33

**Published:** 2016-01-27

**Authors:** Alexander Ivanov, Ambreen Mohamed, Ahmed Asfour, Marc N Katz, Christine Li, Jean Y Ho, Michelle Gorbonosov, On Chen, Joshua Socolow, Sorin Brener, John Heitner

**Affiliations:** 1 grid.415436.10000000404437314Cardiology, New York Methodist Hospital, Brooklyn, NY USA; 2 Cardiology, Maymonides Medical Center, Brooklyn, NY USA; 3 grid.7776.10000000406399286Department of Cardiovascular Medicine, Cairo University, Cairo, Egypt

## Background

Right Atrial Volume Indexed (RAVI) measured by echocardiogram was identified to be an independent predictor of morbidity in patients with heart failure (HF) with reduced ejection fraction (HFrEF). Meta-Analysis Global Group in Chronic heart failure (MAGGIC) risk score is a robust tool in predicting mortality in HF patients. The aim of this study is to evaluate RAVI by cardiac magnetic resonance (CMR) imaging as an independent predictor of all-cause mortality in patients with HFrEF and to compare it with the validated risk score

## Methods

We identify 488 patients with left ventricular ejection fraction (LVEF) < 35% assessed by CMR. We excluded patients undergoing open-heart surgery, severe valvular disease and patients with inadequate imaging. Right atrial volume was calculated based on a measured area in two and four chamber views utilizing a validated equation and indexed to body surface area. MAGGIC risk score was calculated using an online calculator. Follow-up information was acquired via phone call questionnaire and social security death index in patients not able to be reached directly. Our primary outcome was all-cause mortality or an appropriate implantable cardioverter defibrillator therapy.

## Results

Two hundred forty four patients (mean age 60 ± 15; 33% women) were followed for a mean period of 2.1 years. Thirty-two patients (13%) had a primary outcome. The mean RAVI was 53 ± 26 ml/m2. The RAVI was significantly larger in patients with an event than without (75.9 ± 31 ml/m2 vs. 49 ± 23 ml/m2, p < 0.001). The mean MAGGIC score was 19.7 ± 7 (mean one-year mortality 10.2%). RAVI assessed continuously was an independent predictor of mortality controlled for MAGGIC risk, right ventricular ejection fraction (RVEF[JH1]) with HR 1.02(1.01-1.03), p = 0.002. RAVI (0.76 ± 0.08) has a greater C statistic than LVEF (0.55 ± 0.1, p < 0.004), left atrial volume indexed (0.64 ± 0.1, p < 0.025) and has a trend towards greater C statistics than RVEF (0.65 ± 0.11, p < 0.06). The addition of RAVI to the MAGGIC risk score significantly reclassify risk (integrated discrimination improvement by 9%, p < 0.0015 and category-free net reclassification improvement by 58%, p < 0.0029).

## Conclusions

RAVI measured by CMR is an independent predictor of mortality in patients with HFrEF. The addition of RAVI to MAGGIC score significantly reclassify risk.

